# Skin Melanoma and Subsequent Risk of Prostate Cancer: A Lithuanian Cancer Registry Study

**DOI:** 10.3390/ijerph16203915

**Published:** 2019-10-15

**Authors:** Ausvydas Patasius, Vincas Urbonas, Giedre Smailyte

**Affiliations:** 1 Laboratory of Cancer Epidemiology, National Cancer Institute, Santariskiu 1, LT-08660 Vilnius, Lithuania; giedre.smailyte@nvi.lt; 2 Department of Public Health, Institute of Health Sciences of the Faculty of Medicine of Vilnius University, M.K. Ciurlionio 21, LT-03101 Vilnius, Lithuania; 3 Laboratory of Clinical Oncology, National Cancer Institute, Santariskiu 1, LT-08660 Vilnius, Lithuania

**Keywords:** melanoma, prostate cancer, cancer registry, epidemiology

## Abstract

Emerging data indicates that melanoma may be linked to prostate cancer. We evaluated if the incidence of melanoma was associated with subsequent risk of prostate cancer (PC). We extracted data from the Lithuanian cancer registry from 1993 to 2012. We calculated the standardized incidence ratios (SIRs) for PC as a ratio of observed number of cancer cases in people with previous melanoma diagnosis to the expected number of cancer cases in the underlying general population. Therein, 95% confidence intervals for the SIRs were estimated assuming the number of observed cancer cases follows the Poisson distribution. Overall, 65 PCs were observed versus 52.5 expected (SIR 1.24; 95% CI: 0.97–1.58) within a period of 24 years. A significantly increased risk of PC was found in patients with melanoma diagnosis over 70 years (SIR 1.62; 95% CI: 1.11–2.39) and in two periods of diagnosis (SIRs 1.76 and 1.62 in 1993–1997 and 2009–2012, respectively). A significantly increased risk was also found five to nine years after melanoma diagnosis (SIR 1.58; 95% CI: 1.05–2.38). Further studies are needed to evaluate the relationship between melanoma and subsequent risk of prostate cancer.

## 1. Introduction

Melanoma is the most dangerous form of skin cancer and has an hypothesized association with androgens [1]. Steroid hormones, especially androgens, are involved in prostate cancer pathogenesis. Androgen suppression therapy remains one of the main treatment modalities (Bosland, 2000). A number of cancer registry-based epidemiological studies have reported an increased risk of prostate cancer in melanoma patients [2], as well as an increased risk of melanoma after an initial diagnosis of prostate cancer has been shown [3,4,5,6,7].

Both the biological mechanisms and detection biases have been proposed as explanations for the higher rates of melanoma in men with prostate cancer. In a large physician’s health study based on 42,000 men, an increased risk of melanoma was hypothesized to be linked to androgen exposure [5]. In a study from Sweden that used 108,000 men with prostate cancer, it was observed that men had a significantly increased risk of early-stage melanoma if they had a low-risk for prostate cancer, high education, and top income. Therefore, surveillance bias—including PSA (prostate specific antigen) testing, skin examination, and intense sun exposure—have been suggested as risk factors for this association [6].

Skin cancers including melanoma are associated with prolonged exposure to sun [8]. In addition, being the primary cause of melanoma, solar UV (ultraviolet) radiation exposure stimulates the cutaneous synthesis of vitamin D. This presents a clear dichotomy, as vitamin D is treated as a hypothetical anticancer agent [9].

The aim of this population-based cohort study was to determine whether the incidence of prostate cancer in melanoma patients in Lithuania differed from the incidence expected in the general population.

## 2. Materials, Methods, and Results

Data for our study was extracted from the Lithuanian cancer registry, which is a nationwide population-based cancer registry that contains personal and demographic information as well as information on diagnosis of all people diagnosed with cancer in Lithuania since 1978. This analysis was based on the melanoma skin cancers diagnosed in Lithuania between 1993 and 2012. Only patients with their first primary cancer diagnosis within this period were included. The cases were identified from the cancer registry based on the International Classification of Diseases 10th revision (ICD-10) code C43, as well as the International Classification of Diseases for Oncology 3rd edition (ICD-O-3) morphology code 87203–87803. Identified patients were followed until 2016 Dec 31, date of death, lost follow-up, or date of diagnosis of prostate cancer; whichever came first.

We calculated the standardized incidence ratios (SIRs) for prostate cancers as a ratio of observed number of cancer cases in people with previous melanoma diagnosis to the expected number of cancer cases in the underlying general population. Expected numbers were calculated as a multiplication of the exact person-years under observation in the cohort by sex-, year-, and five-year-age-groups, which were specific to the national incidence rates. Moreover, 95% confidence intervals for the SIRs were estimated assuming the number of observed cancer cases follows the Poisson distribution.

Overall, 1693 males were diagnosed with melanoma in Lithuania between 1993 and 2012. Men with a prostate cancer diagnosis prior to their melanoma diagnosis were excluded from the analysis (*N* = 28). Finally, 1665 patients with their first primary cancer of morphologically verified melanoma were examined and followed until the end of 2016 for the second primary prostate cancer, with a total of 9703.6 person-years.

Table 1 presents the observed number of cases, SIRs of developing prostate cancer together with 95% confidence intervals by age, period of melanoma diagnosis, and melanoma diagnosis. Overall, 65 prostate cancers were observed versus 52.5 expected (SIR 1.24; 95% CI: 0.97–1.58) within a period of 24 years. A significantly increased risk of prostate cancer was found in patients with melanoma diagnosis that were over 70 years old (SIR 1.62; 95% CI: 1.11–2.39) and in two periods of diagnosis (SIRs 1.76 and 1.62 in 1993–1997 and 2009–2012, respectively). A significantly increased risk was also found five to nine years after a melanoma diagnosis (SIR 1.58; 95% CI: 1.05–2.38).

## 3. Discussion

Our study provides the population-based evaluation of prostate cancer risk after the first melanoma diagnosis in 1998–2012 in Lithuania with an additional follow-up until the end of 2016. Compared with the general population, the incidence of new prostate cancer after melanoma was higher than expected. However, an observed increase in risk was significant only for melanoma diagnosed in two periods out of four. Our results demonstrated a significantly increased risk of prostate cancers in older melanoma patients and during the period five to nine years after melanoma diagnosis.

This increased risk might be related to environmental factors, behavioral factors, genetic susceptibility, possible shared etiology, or medical surveillance. Prostate cancer and melanoma risk have been described in association with UV exposure. In most studies, higher prostate cancer risk was found in patients with prior melanoma as a marker of extensive sunlight [2]. Older prostate cancer patients have had longer sun exposure throughout their life. Thus, this diagnosis could be associated with the increased risk for the melanoma. Concerning the association between a personal history of melanoma and the risk of other malignancies two opposite effects have been shown. Some studies have shown that melanoma, as a biomarker of vitamin D status, is inversely related with the risk of developing cancers other than melanoma and non-melanoma skin cancers because of the hypothesized anticancer properties of vitamin D [10,11,12]. In other studies, the exact opposite effect was observed, where a personal history of melanoma and non-melanoma skin cancers was associated with the increased risk of other cancers [2,13,14].

In the large Swedish family-cancer database (15.7 million persons), a study twofold increased in risk was observed for melanoma in families with three prostate cancer cases diagnosed. A shared susceptibility for these pairs of cancers was also detected in a study from Iceland [15]. A significantly increased risk of prostate cancer in both first- and second-degree relatives of melanoma cases and increased prostate cancer among the relatives of familial melanoma cases in the Utah population database study was found [16]. In addition, the elevation of melanoma risk among relatives of prostate cancer cases was reported [17]. Findings from Utah are suggestive of heritable cancer syndromes involving predisposition for both melanoma and prostate cancer.

Prostate cancer, as an androgen associated tumor, can potentially be linked to melanoma since it was shown that melanoma cell proliferation was enhanced by testosterone [18,19,20]. Melanocytes produce 5-dihydrotestosterone from androgens [20]. These data indicate that sex hormones might be one of the contributing factors in terms of the association between melanoma and the risk of prostate cancer.

Some authors suggested that the apparent absence of any single environmental agent could explain the multiple cancer risk indicates that the association between skin cancers and risk of other malignancies might be explained by other causes, such as immunosuppression, chronic inflammation, and changes in genomic stability, and that the diagnosis of skin cancer may be a marker of a general high cancer-risk phenotype [13].

The strength of this study is in its population-based nature, which enables comparisons with the same population from which the cases were obtained and avoiding selection bias of the control group. Unfortunately, the registry-based studies are unable to control for the individual level confounders other than sex and age, which limits the strength of the inferences. The main limitation is related to the small size of the study group. Although the follow-up is based on the entire population and the observation period extends 24 years, the number of prostate cancers is very small and thus do not enable statistically robust estimates. Moreover, other limitations of this study should be emphasized. Consequently, the risk pattern for other cancers in such selected group might not be the same as in the general population. Furthermore, there is a risk of surveillance bias. Patients with cancer are under increased surveillance and the possibility to discover another cancer might be higher. In the first year after diagnosis, when melanoma cancer patients have more frequent visits to the doctors and are more likely to undergo additional medical examinations including PSA testing, prostate cancer risk was only slightly insignificantly elevated. The highest risk of prostate cancer in our study was observed in the period five to nine years after diagnosis. This could be interpreted as unaffected by surveillance bias.

## 4. Conclusions

Our results show the association between prostate cancer and melanoma. Despite the ecological study design limitations, we believe that these findings provide the evidence concerning a potential link between melanoma and prostate cancer. Future research is needed to further clarify the relationship between melanoma and prostate cancer and to determine the underlying mechanisms of those disease development.

## Figures and Tables

**Table 1:** Numbers of observed (Obs) cases of prostate cancers, standardized incidence ratios (SIR) with 95% confidence intervals (CI) in melanoma patients.

Variables	Obs	Exp	SIR	95% CI		*P* Value
All melanoma patients	65	52.49	1.24	0.97	1.58	0.08
Age, years						
<60	17	15.5	1.10	0.68	1.76	0.70
60–69	22	20.98	1.05	0.69	1.59	0.82
70+	26	16	1.62	1.11	2.39	0.01
Period of diagnosis						
1993–1997	14	7.94	1.76	1.04	2.98	0.03
1998–2002	9	14.88	0.61	0.31	1.16	0.15
2003–2008	31	19.17	1.62	1.14	2.30	0.01
2009–2012	11	10.50	1.05	0.58	1.89	0.88
Time after diagnosis, years						
<1	8	0.7	11.43	5.71	22.85	<0.01
1–4	22	7.93	2.77	1.83	4.21	<0.01
5–9	23	15.22	1.51	1.00	2.27	0.05
10+	12	28.64	0.42	0.24	0.74	<0.01
